# Cardiorespiratory Fitness and Health-Related Quality of Life in Secondary School Children Aged 14 to 18 Years: A Cross-Sectional Study

**DOI:** 10.3390/healthcare10040660

**Published:** 2022-03-31

**Authors:** Lidija Marković, Jovana Trbojević Jocić, Maja Horvatin, Damir Pekas, Nebojša Trajković

**Affiliations:** 1 Faculty of Sport and Physical Education, University of Novi Sad, 21000 Novi Sad, Serbia; markoviclidija169@gmail.com; 2 Department of Social Science, Matica Srpska, 21101 Novi Sad, Serbia; jovana.trbojevic88@gmail.com; 3 Faculty of Kinesiology, University of Zagreb, 10000 Zagreb, Croatia; maja.horvatin@kif.unizg.hr (M.H.); damir.pekas@kif.unizg.hr (D.P.); 4 Faculty of Sport and Physical Education, University of Niš, 18000 Niš, Serbia

**Keywords:** cardiorespiratory fitness, health-related quality of life, BMI, adolescents, secondary school children

## Abstract

The main aim of this study is to examine age and gender differences in cardiorespiratory fitness (CRF) among Serbian secondary school children. The secondary aim is to explore the association between CRF and quality of life in Serbian adolescents. The sample consisted of 579 adolescents (285 males), aged from 14 to 18 years old. To evaluate their anthropometric measurements, body height and body weight were examined, the 20 m shuttle run test was used to access CRF, and the standardized global measure of wellbeing KIDSCREEN was used to access the wellbeing of adolescents. The results show that the boys possessed higher CRF compared to the girls, as well as higher scores on variable distance, but there were no significant differences according to age. CRF was positively associated with physical wellbeing, psychological wellbeing, total score HRQL, body height and body weight, and negatively correlated with BMI. Conversely, physical wellbeing showed positive correlation with the other subscales of KIDSCREEN (psychological wellbeing, autonomy and parents, peers and social support, and school environment), and total score of (Health–Related Quality of Life) HRQL. The results showed that better CRF would be beneficial for quality of life among Serbian adolescents, especially among girls. Moreover, the relationship between CRF and BMI shows that adolescents with regular values of BMI have better physical fitness and wellbeing.

## 1. Introduction

With the development of technology and modern society, the last decade has seen a declining percentage of young people participating in some form of physical activity. Although organized sports are one of the most frequent activities for young people after school, the trend of dropping out of sports in recent years is growing rapidly in European countries and the world. Namely, on average, 17 to 35% of young people annually drop out of playing sports during adolescence [1,2]. In Serbia there are similar data, where the research conducted in 2017 showed that about 11% of young people annually drop out of organized sports [3]. When the focus shifts to general physical activity among young people, the same trend is observed. Namely, according to data from five European countries, only 4.6% of girls and 16.8% of boys complied with the 60 min/day of moderate- to vigorous-intensity physical activity daily [4,5]. According to the data of the Republic Institute for Sports and Sports Medicine of the Republic of Serbia from 2015, 56% of boys and 75% of girls do not meet the minimum recommended amount of physical activity [6].

Physical fitness covers all the physical qualities of a person and could be an integrated part of all the functions and structures involved in the performance of physical exertion [7]. Several earlier studies regarding health-related physical fitness [8,9,10,11] have recognized body fatness, as well as cardiorespiratory fitness, as health-related fitness traits [11]. One of them remains the leading cause of death in adults worldwide [12,13]. Although cardiovascular diseases (CVD) appear during late adulthood, there is plenty of evidence that the disease originates in early life [14,15,16,17,18]. For instance, higher blood pressure and high body mass index (BMI) in childhood predict poorer cardiovascular health in adulthood [19,20]. In addition, poor physical fitness could have a greater influence than other well-established factors, such as dyslipidemia, hypertension, or obesity [21]. Conversely, a high level of physical fitness is associated with a reduction in CVD risk factors [21,22], and is potentially a useful predictor of CVD risk factors and overall health [23]. The investigation of these factors could be relevant for the diagnosis and prevention of many diseases in adults.

Cardiorespiratory fitness (CRF) is a structural part of health-related physical fitness. The related developmental patterns of CRF in gender and age have been well studied [24]. The estimation of VO_2max_ is based on the linear association between heart rate and VO_2_ used in several submaximal exercise tests [25], with the 20 m shuttle run test the most commonly used. This test evaluates the maximum aerobic capacity based on an indirect-incremental-maximum field test involving a 20 m shuttle run, using the specific formulas for calculating the maximal oxygen consumption (VO_2max_) [26]. The timing and tempo of growth and maturation influence an individual level of VO_2max_, and comparing it to normative values for the general population displays a full image of the development of CRF in adolescence.

The previous study of age- and gender-specific percentiles for U.S. youth aged 12–18 years was based on the National Health and Nutrition Examination Survey (1999–2002), and the recent application of the LMS (L = skewness, M = median, and S = coefficient of variation) statistical procedure for the construction of growth percentiles was added for a variety of outcomes, and relations were made to current FITNESSGRAM^®^ thresholds. The results showed that the growth curves for boys are similar to previous research on developmental changes in CRF. There was a slight increase in estimated VO_2max_ of boys aged 12–15 years, and then it remained relatively stable. Conversely, there was a slight decrease in estimated VO_2max_ across ages 12–18 years in girls, which displayed higher values at every age-specific percentile in boys [27].

However, in addition to the important role of fitness on the physical health of children and adolescents, an increasing number of researchers are paying attention to the effects of fitness on the psychological wellbeing of young people. Thus, research has shown that physical activity in childhood and adolescence contributes to the development of cognitive processes [28,29], of physical and personal self [30,31], socioemotional skills, prosocial behavior [27,32], self-regulation, leadership skills, and better mental health [33].

The relation of physical fitness in childhood/adolescence with physical health and wellbeing in adulthood has been noted by several studies [34], which is why researchers have shifted their attention to adolescence as a risk period for declining physical activity. In addition to physical activity itself, in recent years special attention has been paid to the Health-Related Quality of Life (HRQL) of adolescents used to assess youth health and identify young people with various chronic diseases [35] and health-risk behaviors [36]. Moreover, an increasing number of studies show that cardiorespiratory and muscular fitness are associated with higher HRQL in children and adolescents [37,38].

These relations indicate that being physically active and able can contribute to feeling competent, having a positive self-image, and also being viewed as competent by others—and all of these segments can influence social and emotional development and integration in peer groups, and as a final result have a greater impact on quality of life. To determine the relation between aerobic fitness and quality of life in adolescence, it was important to implement the systematic measurement of adolescent wellbeing on a public health basis [39,40]. The standardized global measure of wellbeing, KIDSCREEN, according to the European consortium, available in 38 languages, in 10-, 27-, and 52-item versions [41,42] has been used to access the wellbeing of adolescents

Many studies, mostly in Europe, but also in Africa, Asia, and South America, have incorporated different versions of KIDSCRREEN: the version of KIDSCREEN-10 among school-aged children [43]; the parent-rating version of KIDSCREEN-10 in 27 EU countries [10,38], and the self-report version in a sample of children with cerebral palsy in KIDSCREEN-52; on their own or with parental help [44]. Based on results, systematically developed KIDSCREEN questionnaires measured equivalent dimensions in wellbeing across a variety of cultures, allowing further monitoring and comparing differences between adolescent trends for national and international data [44].

A study from Norway on a sample of preschool children applied KIDSCREEN-27 to assess wellbeing in order to examine the association between physical activity and HRQL [45]. In their research, it was pointed out that there was a moderate positive relationship between the degree of physical activity and the dimension of physical wellbeing from KIDSCREEN-27 [45]. In a sample of Brazilian adolescents aged 12 to 17, where KIDSCREEN-52 and physical fitness tests were applied (handgrip strength test, flexibility test, and cardiorespiratory fitness test), found that adiposity and cardiorespiratory fitness contribute to the physical wellbeing dimension of HRQL [34].

The main aim of this study is to examine age and gender differences among Serbian secondary school children in CRF and the distances that students ran in the shuttle run test. Furthermore, since there was the connection between CRF and quality of life in the previous research, the secondary aim is to explore the association between CRF and HRQL in Serbian adolescents.

## 2. Materials and Methods

### 2.1. Participants

The current study was conducted in 2019 in order to examine aerobic fitness in Serbian secondary school children, and how it affects their quality of life. The sample consisted of 579 adolescents (285 males), aged from 14 to 18 years old. They were secondary school students and were involved in regular physical education (PE) classes two times per week. The study was conducted by a team of researchers with experience in carrying out the measures, and all procedures were approved by the Faculty of Sport and Physical Education Ethical board (No. 27/2019). Teachers and school administrators were fully informed about all study procedures and signed informed consent for the measurements. Verbal consent from the child and their parents to participate in the study was necessary on the measurement day.

### 2.2. Procedures

To evaluate their anthropometric measurements, body height and body weight were examined by trained field examiners (physical education teachers). Children were measured in gym clothes without their shoes and heavy objects. Bodyweight was measured to the nearest 0.1 kg with portable digital scales (Omron BF214). Body height was measured to the nearest 0.1 cm with a stadiometer (Seca 213).

The multistage fitness test (the 20 m shuttle run test) was used to access cardiorespiratory fitness during their PE classes. According to the number of students in class and the professor’s list from the school diary, students were divided into several groups (10 to 15 students in each group). The number of total laps was recorded, and every stage from 0.5 to 20 defined different speeds and number of laps. It was obligatory to run every lap between two sound signals, and they had one chance to miss it before they finished the test. The value of VO_2max_ and distance (total distance in meters that students ran) were used for further analysis. The value of VO_2max_ was calculated according to a decimal score of running laps by formula VO_2max_ = 31.025 + 3.238 (S) 2 3.248 (A) + 0.1536 (A 3 S), where S = speed in kilometers per hour at the end of the test, and A = age in years [26].

To assess the quality of life in Serbian adolescents, KIDSCREEN 27 for children and young people was used. The KIDSCREEN generic health-related quality of life measure for children and adolescents was developed within a European project “Screening and Promotion for Health-related Quality of Life in Children and Adolescents—A European Public Health Perspective” funded by the European Commission [38].

KIDSCREEN-27 was developed to construct a shorter version of KIDSCREEN-52. KIDSCREEN-27 consists of a 5-dimensional structure: Physical Wellbeing (5 items), Psychological Wellbeing (7 items), Autonomy and Parents (7 items), Peers and Social Support (4 items), and School Environment (4 items). Each item is scored on a 5-point scale (1 = “not at all”, 2 = “a little”, 3 = “moderately”, 4 = “much”, and 5 = “very much”). Certain items are reversed when scoring the questionnaire. For each dimension, a scoring algorithm is used to calculate T-scores scaled with a mean of 50 and a standard deviation of 10, while the total KIDSCREEN score is generated by summing up all item responses. Higher scores indicate better HRQL [38,41]. Students had to fill in the questionnaire after they finished their shuttle run test. Students answered questions related to physical activity and health, general mood and feeling about yourself, family and free time, friends, school and learning during the past week, and general health.

These measurements were conducted before the COVID-19 pandemic, so there was no possible influence on the results.

### 2.3. Statistics

For all data, the descriptive statistics were calculated as mean and standard deviation (±SD). Normality of data was assessed using the Kolmogorov–Smirnov test. For examining gender differences in the degree of aerobic endurance and distance, a *t*-test was performed for independent samples; a covariance analysis was conducted for investigating the roles of gender, with height, and weight as covariance in possible differences in the degrees of aerobic endurance and distance. For examining age differences, covariance analysis was conducted with gender, height, and weight as covariance in possible differences in the degrees of aerobic endurance and distance. Linear regression analysis was conducted in order to further examine the relationship between CRF, Health-Related Quality of Life (HRQL), gender, and age. To examine if there was a significant interaction effect of gender and age group on CRF and distance, two-way ANOVA was performed (with height and weight as control variables). In order to examine relations between CRF and wellbeing in adolescence, correlation analysis was conducted. All statistical analysis was performed using IBM software (SPPS Inc. 24, Chicago, IL, USA). Statistical significance was set on *p* ≤ 0.01.

## 3. Results

The sample consisted of 579 adolescents, aged 14 to 18 years (mean = 16.58)—285 males and 294 females. Table 1 provides additional sample descriptions.

### 3.1. Gender Differences in CRF and Distance

The results of the *t*-test show that the boys achieved statistically significantly higher results on the 20 m shuttle run test (t(455) = 15.13, *p* ≤ 0.001), i.e., possessed higher CRF compared to girls (Table 2). Boys had statistically higher scores on variable distance (t(437) = 15.59, *p* ≤ 0.001) when compared with girls. The covariance analysis confirms the results of the *t*-test that there were significant gender differences in the degree of CRF (F(3578) = 114.05, *p* ≤ 0.001) and distance (F(3578) = 118.38, *p* ≤ 0.001) when height and weight were taken as a control variable.

### 3.2. Age Differences in CRF and Distance

In order to examine the age differences in the degree of CRF, the participants were divided into five age categories (14, 15, 16, 17, 18), after which a one-way analysis of variance was performed. Height, weight, and gender were taken as control variables.

The results show that there are no statistically significant age differences in the degree of CRF and distance (Table 3).

What can be seen from Table 3 is that although no significant differences were found, adolescents aged 15 had the highest CRF (Mean = 29.45), and adolescents aged 14 had the lowest (Mean = 27.04).

To see if there was a statistically significant interaction effect of gender and age group on CRF and distance, two-way ANOVA was performed (with height and weight as control variables). The results show that effect of interaction of gender and age group on CRF (F(11,579) = 0.192, *p* = 0.943) or distance (F(11,579) = 0.237, *p* = 0.917) was not significant.

### 3.3. Relations between CRF and HRQL in Adolescence

In order to examine relations between CRF and HRQL in adolescence, correlation analysis was conducted. As relevant variables, height, weight, BMI, gender, and age were included with VO_2max_ and t scores of five subscales of the KIDSCREEN generic HRQL measure (physical wellbeing, psychological wellbeing, autonomy and parents, peers and social support, and school environment), and total score of HRQL.

CRF was moderately positively and significantly correlated with physical wellbeing (r = 0.509, *p* < 0.010), and height (r = 0.434, *p* < 0.010), and had a lower but positive and significant correlation with psychological wellbeing (r = 0.092, *p* < 0.050), total score HRQL (r = 0.198, *p* < 0.010), and body weight (r = 0.199, *p* < 0.010); and a negative correlation with gender (r = −0.560, *p* < 0.010) and BMI (r = −0.086, *p* < 0.050). On the other hand, physical wellbeing showed a positive correlation with psychological wellbeing (r = 0.318, *p* < 0.010), autonomy and parents (r = 0.225, *p* < 0.050), peers and social support (r = 0.170, *p* < 0.010), school environment (r = 0.239, *p* < 0.010), and total score of HRQL (r = 0.572, *p* < 0.010); and a negative correlation with gender (r = −0.270, *p* < 0.010) and age (r = −0.142, *p* < 0.010). There is a significant positive intercorrelation between the five subscales of KIDSCREEN and total score (Table 4).

In order to further examine the relationship between CRF and wellbeing, regression analysis was performed for the total score HRQL. Gender, age, and VO_2max_ were considered as predictors.

Results show that this predictive model is statistically significant (F(3578) = 11.203, *p* ≤ 0.001) and explains 5% of variance of HRQL.

As first, the individual significant predictor of HRQL aerobic endurance was singled out, and then age. Results show that higher aerobic endurance contributes to better quality of life, and that perceived quality of life declines with age (Table 5).

## 4. Discussion

Having in mind the importance of physical activity in terms of the overall development of individuals and society, this study takes the adolescent period as one of the key age periods for further participation in physical activity in order to examine gender and age differences in CRF and distance, and the relation of CRF to HRQL.

In accordance with previous research [7,46], significant gender differences in CRF and distance were noted. The boys achieved statistically significantly higher results on the 20 m shuttle run test, as well as on variable distance, compared to girls, when height and weight were taken as covariate variables.

**Figure 1:**
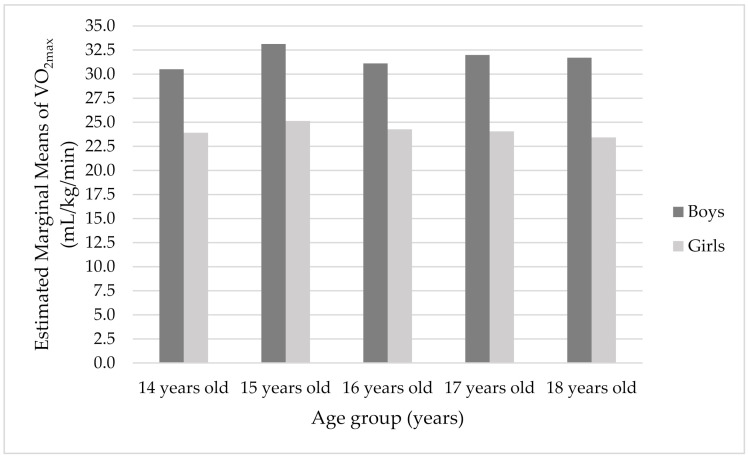
The plot of the mean VO_2max_ score (mL/kg/min) for each combination of groups of gender and age group. Covariates appearing in the model are evaluated at the following values: Height (cm) = 172.828, Weight (kg) = 66.202.

**Figure 2:**
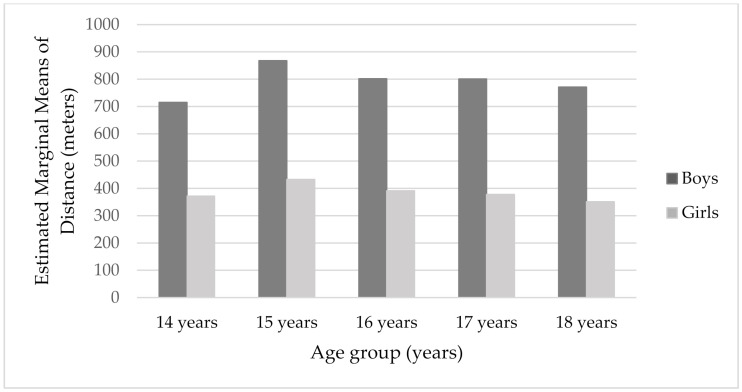
The plot of the mean distance score (meters) for each combination of groups of gender and age group. Covariates appearing in the model are evaluated at the following values: Height (cm) = 172.828, weight (kg) = 66.202.

On the other hand, no significant age differences in CRF and distance were obtained in this study when gender, height, and weight were taken as covariance. However, when we look at the results of the two-way ANOVA and the achieved scores by age groups, it can be seen that the age of 15 is the peak of CRF for both boys and girls, and stably declines for boys with age; but declines, then slightly increases at age 17, and stably declines with age for girls (Figure 1). The same trend can be observed in distance measure (Figure 2). The lowest CRF was shown at age 14. CRF is a very important component of children’s health [8,17]. In addition, children and adolescents’ CRF is related to many health outcomes, and consequently with many chronic diseases in adults [8,17]. The estimation of CRF was evaluated by the 20 m shuttle run test. Normally, children and adolescents’ peak VO_2_ increases through growth and maturation, although there are indications of girls’ peak VO_2_ being reached at around age 14 [46]. In general, higher values of peak VO_2_ have been noted in boys [6,46], and sex differences are enhanced as they progress through adolescence. These could be attributed to boys’ greater muscle mass and hemoglobin concentration [46]. Moreover, it could be explained by a slight increase in body fat in boys between 7–12/13 years of age and a reduction in body fat in puberty [36]. These current findings showed the lowest CRF at age 14, but a peak at age 15. After a period of peak values, there was a slight decline in boys, but there was a small increase at girls evident at around age 17, which stably declined after. Similarly, Eisenmann et al. (2011) discovered a slight increase in estimated VO_2max_ of boys aged 12–15 years, which then remained stable. Furthermore, there was a slight decrease in estimated VO_2max_ across ages 12–18 years in girls.

Research that has examined the relationship between physical activity and psychological wellbeing largely shows a positive trend between these two constructs, i.e., that physical activity contributes to the psychological wellbeing of young people [18,24]. In this study, we wanted to examine whether there is a link between CRF and adolescent wellbeing. The obtained results show that CRF, together with gender and age, represent a significant predictor model that explains 5% of adolescent wellbeing, i.e., health-related quality of life. CRF and age stood out as significant individual predictors. Better CRF contributes to health-related quality of life, while age has the opposite effect, i.e., with age, the health-related quality of life decreases. Although the percentage of explained variance is small, in order to better understand the relationship between CRF and health-related quality of life, we can look at the correlation results (Table 4). CRF is positively associated with physical wellbeing (subscale of the overall HRQL score). However, we can also observe that the subscale of physical wellbeing is significantly associated with other subscales (psychological wellbeing, autonomy and parents, peers and social support, and school environment). This relationship indicates the possibility of an indirect effect of CRF on the overall wellbeing of adolescents. Namely, adolescents who are more physically active can develop a positive image of themselves through sports, and gain a sense of competence, which is an important factor in forming interpersonal relationships, personal identity, and adjustment to the environment [47]. Physical fitness is one of the factors that plays a significant role in adolescence and acceptance by peers [48]. Based on the obtained results, CRF can be viewed as a protective factor of health-related quality of life in adolescence.

The association between HRQL and age has also been found in previous research [49]. Given that adolescence is a risky age period in terms of the frequency of physical activity—that physical activity decreases with age—it is not surprising that HRQL itself decreases with age. The course of adolescence implies pronounced physiological, physical, social, emotional, and cognitive changes, as a result of which adolescence represents an age period with an increased risk of developing mental health problems [49]. Life satisfaction declines with age due to the challenges and developmental challenges facing adolescents, for which adolescents may not yet have adaptive strategies. When we look at the decline in physical activity, which has the role of a protective factor in mental health, then the result that HRQL decreases with age further invites us to continue to deal with the relationship between physical activity and life satisfaction in adolescents.

This is the first study in this region that examined the correlation between CRF and HRQL. It is very important to assess wellbeing among children and adolescents, as well as certain domains of physical fitness. CRF was evaluated by the 20 m shuttle run test. Further investigation should focus on measuring CRF by direct measures, which will provide a more complete and valid description of the power–duration relationship than performance in a single test. Given the importance of CRF on adolescent wellbeing, future research could focus on even younger children, as well as the association of CRF with prosocial behavior, degree of adaptation in social groups, and longitudinal effects of CRF in childhood and adolescence on physical activity in adulthood. Bearing in mind that there are significant gender differences in the degree of CRF, as well as that CRF is a protective factor, it is necessary to further investigate the factors that affect the reduction of CRF in girls.

## 5. Conclusions

The boys possessed higher CRF compared to girls, as well as higher scores in covered distance, but there were no significant differences according to age. Both boys and girls had their peak for CRF at the age of 15, which stably declines for boys with age, and for girls after age of 17.

CRF showed a moderate positive correlation with physical wellbeing, body height, and total score HRQL; and a lower but positive correlation with psychological wellbeing and body weight. Conversely, CRF was negatively associated with age and BMI. Furthermore, physical wellbeing was positively associated with the other subscales of KIDSCREEN and total score of HRQL.

CRF and age represent significant predictors of HRQL. Higher CRF can contribute to better HRQL. Examination of age and gender differences in CRF among Serbian secondary school children, and the associations between CRF and HRQL, suggest that there is an urgent need for an increase in engagement within PE classes or through some recreational activities.

## Figures and Tables

**Table 1:** Sample structure by gender and age.

Age	Boys (*n* = 285)	Girls (*n* = 294)
14 (*n* = 67)	33	34
15 (*n* = 136)	72	64
16 (*n* = 135)	57	78
17 (*n* = 139)	67	72
18 (*n* = 102)	56	46
Height (cm)	179.57 (7.38)	166.29 (6.69)
Body weight (kg)	71.78 (12.05)	60.79 (10.09)
BMI	22.22 (3.32)	21.96 (3.43)

Note: Values are mean (S.E).

**Table 2:** Results of *t*-test for gender differences in CRF and distance.

		**Mean**	**Std. Deviation**	***t*-Test (df)**	***p***
VO_2max_	Boys	32.26	7.93	15.13 (455)	0.001
	Girls	24.06	4.64		
distance	Boys	814.46	417.41	15.59 (437)	0.001
	Girls	376.53	228.70		

**Table 3:** Results of ANOVA for age differences in CRF and distance.

		Mean	Std. Deviation	F (df)	*p*
VO_2max_	14	27.04	7.139	1.382 (7578)	0.239
	15	29.45	8.104		
	16	27.66	7.708		
	17	27.99	7.556		
	18	27.70	7.347		
Distance	14	533.73	373.49		
	15	666.62	420.04	1.639 (7578)	0.163
	16	568.30	409.30		
	17	588.06	389.54		
	18	568.04	385.70		

**Table 4:** Correlations between VO_2max_, 5 subscales of the KIDSCREEN, total score of HRQL, anthropometric measurements, gender, and age of secondary school children.

	1	2	3	4	5	6	7	8	9	10	11	12
1. VO_2max_	*	0.509 **	0.092 *	0.031	0.005	0.013	0.198 **	0.434 **	0.199 **	−0.560 **	−0.017	−0.086 *
2. physical wellbeing		*	0.318 **	0.225 **	0.170 **	0.239 **	0.572 **	0.223 **	0.082 *	−0.270 **	−0.142 **	−0.070
3. psychological wellbeing			*	0.474 **	0.403 **	0.422 **	0.792 **	0.031	−0.027	−0.061	−0.084 *	−0.062
4. autonomy and parents				*	0.409 **	0.438 **	0.748 **	0.052	−0.016	−0.082 *	−0.125 **	−0.059
5. peers and social support					*	0.234 **	0.577 **	−0.002	−0.012	0.010	0.029	−0.004
6. school environment						*	0.654 **	−0.072	−0.117 **	0.050	−0.192 **	−0.098 *
7. HRQL							*	0.072	−0.021	−0.111 **	−0.160 **	−0.082 *
8. Height								*	0.630 **	−0.723 **	0.129 **	0.051
9. Weight									*	−0.477 **	0.170 **	0.781 **
10. Gender										*	−0.025	−0.062
11. Age +											*	0.119 **
12. BMI												*

+ age as continued variable, not as category * *p* < 0.050. ** *p* < 0.010.

**Table 5:** Relations between CRF and HRQL according to gender, age and VO_2max_.

	Unstandardized Coefficients		Standardized Coefficients Beta	t	Sig.	Correlations			Collinearity Statistics	
	B	Std. Error				Zero-Order	Partial	Part	Tolerance	VIF
(Constant)	119.167	8.289		14.377	0.001					
vo_2max_^a^	0.297	0.081	0.175	3.650	0.001	0.186	0.150	0.148	0.712	1.405
gender ^b^	−0.338	1.245	−0.013	−0.272	0.786	−0.104	−0.011	−0.011	0.712	1.405
age *^c^	−1.560	0.437	−0.145	−3.568	0.001	−0.148	−0.147	−0.145	0.998	1.002

^a, b, c^ predictors of dependent variable HRQL. * age as continued variable, not as category.

## Data Availability

The data presented in this study are available from the corresponding author upon request.
